# Tuberculosis deaths: are we measuring accurately?

**DOI:** 10.1186/2052-3211-7-16

**Published:** 2014-11-14

**Authors:** Muhammad Atif, Syed Azhar Syed Sulaiman, Asrul Akmal Shafie, Muhammad Qamar Uz Zaman, Muhammad Asif

**Affiliations:** Department of Pharmacy, The Islamia University of Bahawalpur, Punjab, Pakistan; Discipline of Clinical Pharmacy, School of Pharmaceutical Sciences, Universiti Sains Malaysia, Penang, Malaysia; Discipline of Social and Administrative Pharmacy, School of Pharmaceutical Sciences, Universiti Sains Malaysia, Penang, Malaysia; Department of Pharmacology, School of Pharmaceutical Sciences, Universiti Sains Malaysia, Penang, Malaysia

## Abstract

Death among tuberculosis patients is one of the major reasons for non-attainment of 85% treatment success target set by World Health Organization. In this short paper, we evaluated whether the overall mortality rate in pulmonary tuberculosis is being affected by other comorbid conditions. All new smear positive pulmonary tuberculosis patients (N =336), who started their treatment at the chest clinic of the Penang General Hospital, between March 2010 and February 2011, were followed-up until December 2011. Tuberculosis treatment outcomes were reported according to six treatment outcome categories recommended by World Health Organization. The outcome category ‘died’ was defined as ‘a patient who died due to tuberculosis or other cause during tuberculosis treatment’. Our findings showed that out of 336 smear positive pulmonary tuberculosis patients, 59 (17.6%) died during treatment (mortality rate = 1.003 cases per 1000 person-days of follow-up).

Among the deceased patients, the mean age was 55.8 years (SD =16.17) and 49 were male. According to the mortality review forms, 29 deaths were tuberculosis-related, while the remaining 30 patients died due to reasons other than tuberculosis. Cerebrovascular accident (n =7), septicaemia shock (n =4) and acute coronary syndrome (n =4) were the most common non-tuberculosis related reasons for mortality in the patients. If the 30 patients, for whom tuberculosis was incidental to death, are excluded from the final cohort, the proportion of patients in the ‘died’ outcome category could be reduced to 9.5%. The treatment outcome criterion (i.e., died) set by World Health Organization has limitations. Therefore, it requires improvement for more objective evaluation of the performance of the National Tuberculosis Program.

## Background

Outcomes of tuberculosis (TB) treatment are reported in line with six outcome categories (i.e., cured, treatment completed, treatment failure, defaulter, died and transferred out) recommended by World Health Organization (WHO) [1]. Cured and treatment completed are further categorized as successful treatment outcomes, while the latter four represent unsuccessful treatment outcomes [2–4]. The National Tuberculosis Program (NTP) of every country identifies the key areas for improvement. However, appropriate corrective actions are sometimes futile due to reasons which are beyond the program’s control within a specific context.

According to the standard guidelines [1], a person who dies due to any reason during TB treatment is included in the ‘died’ category. From this definition, it is evident that even patients for whom TB is incidental to death rather than causal should also be classified in the ‘died’ category of treatment outcomes. For example, if a TB patient dies of cancer during TB treatment, he or she should be declared as died due to TB. In such a case, even if the NTP staff takes timely and appropriate actions, these deaths are not preventable.

Most of the earlier studies on treatment outcome of TB explained patients’ death as one of the major causes of non-attainment of the 85% target treatment success rate [5–7]. In this paper, we evaluated whether the overall mortality rate in the patients with pulmonary tuberculosis is being affected by the other comorbid conditions (Note: The findings presented in this commentary are part of our previously published primary data [7] for which the publication approval has been obtained from the Director General of Health, Malaysia). The study was approved by the Medical Research Ethics Committee (MERC), Ministry of Health, Malaysia (Registration ID: NMRR-10-77-5099; MERC reference: dim. KKM/NIHSEC/08/08/04P10-69).

## Methods and key findings

All new smear positive PTB patients who received standardized anti-TB therapy [1] at the chest clinic of the Penang General Hospital, Malaysia, between March 2010 and February 2011, were followed-up until December 2011. A detailed description of the study setting has been published [7]. Medical charts and TB notification forms of the patients were reviewed to obtain socio-demographic and clinical data. Treatment outcomes of the patients were reported according to six outcome categories as stipulated in the WHO guidelines. The treatment outcome category ‘died’ was defined as ‘a patient who died due to TB or other cause during TB treatment’ [1]. A mortality review panel which comprised of the medical doctors including chest physicians evaluated the cause of the patient’s death. The decision, whether the patient died due to TB or other reasons was based on available laboratory data, *post mortem* reports and/or clinical judgment of the medical doctors.

The mortality rate by cause in our cohort was calculated by dividing the number of deaths by person-days at risk calculated from the time treatment initiated until the death or end of the treatment [8].

Out of 336 patients registered during the study period [7], 59 (17.6%) died during their TB treatment (mortality rate = 1.003 cases per 1000 person-days of follow-up). Among the deceased patients, the mean age was 55.80 years (SD =16.17), 49 were male, 41 were married, 28 were diabetics, 31 were alcoholics, 39 were smokers, eight were drug abusers and four were positive for Human Immunodeficiency Virus (HIV). Thirty-five patients died within the first month, whereas eight, nine and seven patients died during the second, fourth and sixth month of their TB treatment, respectively. Only four patients died at home, whereas the remaining 55 patients died at the hospital.

According to the mortality review forms, which were completed by a panel of physicians including a senior chest consultant, 30 out of 59 patients died due to reasons other than TB (Table 1).

**Table 1:** **Primary causes of death in the patients**

Causes of death	Patients n (%)
**Tuberculosis**	**29 (49.2)**
**Non-tuberculosis**	**30 (50.8)**
Cerebrovascular accident	7 (11.9)
Septicemia shock	4 (6.8)
Acute coronary syndrome	4 (6.8)
Pulmonary embolism	2 (3.4)
Advanced retroviral disease	2 (3.4)
Acute exacerbation of COPD^*^	2 (3.4)
Other	9 (15.3)
**Total**	**59**

^*^COPD: Chronic obstructive pulmonary disease.

In this study, the proportion of deceased patients (17.6%) was similar to that reported in some of the earlier studies from the United States and Western Pacific Region [9–11]. Two European studies reported that a higher death rate among TB patients was one of the major reasons for not meeting the 85% TB treatment success target [5, 6]. In our study, besides expected default, transfer out and treatment failure rates, the death rate of more than 15% is alone responsible for not meeting the success target of 85%. However, if the 30 patients, for whom TB was incidental to death, are removed from final analysis, the death rate could be reduced to 9.5% (29/306; mortality rate = 0.493 cases per 1000 person-days of follow-up).

Certainly, these findings will not only allow the NTP managers to have a more objective evaluation of the NTP, but also increase the overall treatment success rate in a given cohort. A Russian study also stated that using such deaths, in which TB is not the actual cause of death, as an indicator of NTP’s performance might be misleading, and these deaths might not have been prevented even with improvements in the TB services [8]. In a similar fashion, a UK study demonstrated that it is unreasonable to consider such deaths as an unsuccessful treatment outcome of TB [12].

Of course, one might think that if 30 patients, for whom TB was incidental to death, cannot be placed in the ‘died’ outcome category of TB treatment outcomes, then they should be classified somewhere. Unfortunately, the current TB treatment outcome categories are unable to accommodate such patients separately. However, the UK modified criteria for monitoring TB clinical outcomes classify such patients in the successful treatment outcome category [12]. Classifying the treatment outcome of such patients as treatment success may be controversial as these patients died, which is not the target of any treatment modality.

## Conclusion

The tuberculosis treatment outcome criterion (i.e., died) set by the WHO has limitations, and requires improvements for a more objective evaluation of the performance of the NTP. We suggest that TB patients for whom TB is not the actual cause of death should be categorized in a separate outcome category, possibly ‘died not due to tuberculosis’. It is further suggested that such patients should neither be categorized in the successful treatment outcome category nor in the unsuccessful treatment outcome category.
